# Supplementary material for: Comparison of Self-Reported and Device-Based Measured Physical Activity Using Measures of Stability, Reliability, and Validity in Adults and Children
Source: Sensors (Basel). 2021 Apr 10;21(8):2672. doi: 10.3390/s21082672 (PMC8069485; doi:10.3390/s21082672)
Supplement: Supplementary file 1 [file sensors-21-02672-s001.pdf]

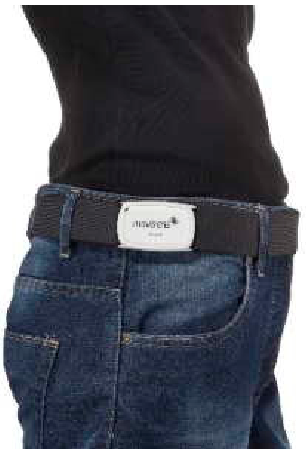

Figure S1. Move 4 (Movisens GmbH, Karlsruhe, Germany) sensor attached to the right hip of a participant (*Move 4 | movisens Docs*. Available at: https://docs.movisens.com/Sensors/Move4/#attaching-the-sensor-to-the-test-person; 2021 [accessed 16.03.2021]).

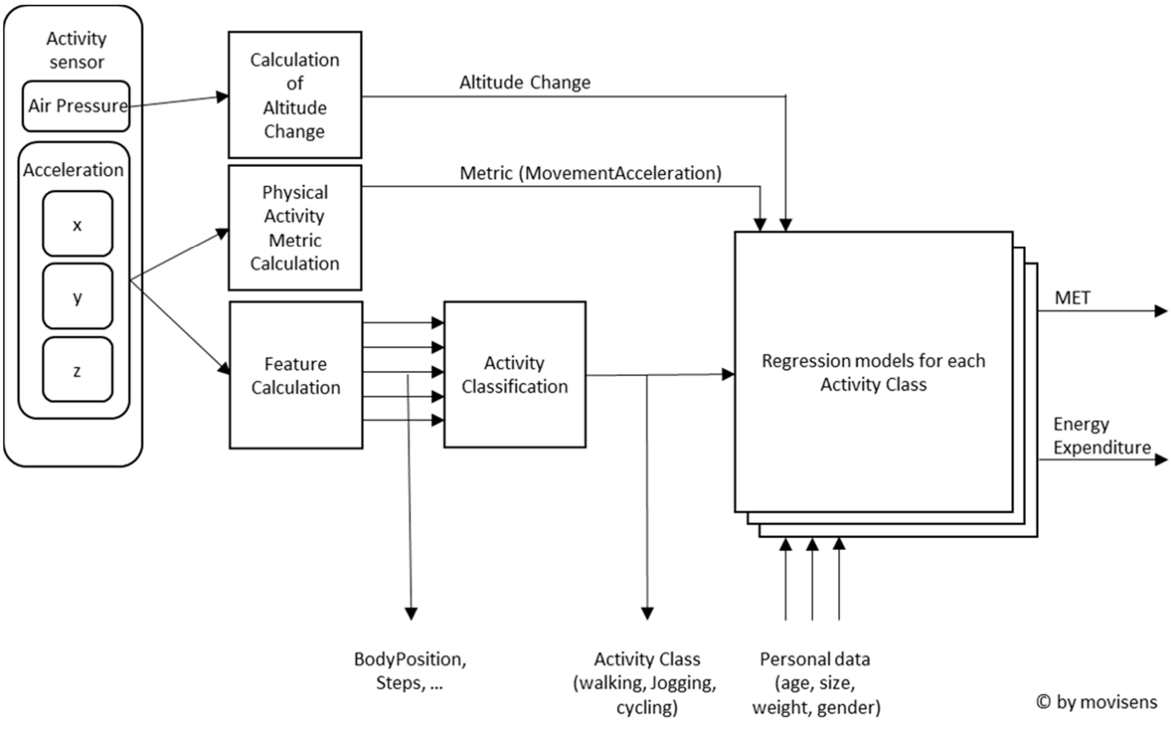

Figure S2. Energy expenditure estimation. Displayed is the decision path of energy expenditure and metabolic equivalent (MET) estimation based on acceleration and air pressure measurements of movisens sensor and personal data of the participants (*Energy expenditure | movisens Docs*. Available at: https://docs.movisens.com/Algorithms/energy_expenditure/#metabolic-equivalent-of-task-met; 2020 [accessed 21.12.2020]).

Table S1. Descriptive data of moderate (MPA) and vigorous (VPA) physical activity of adults. Displayed are the number of participants (n), means and standard deviations (SD) at two measurements at least three weeks apart (T_0 and T_1) for the parameters accelerometry with 10 seconds epoch length (Acc 10) and 60 seconds epoch length (Acc 60), physical activity diary (diary), the International Physical Activity Questionnaire (IPAQ), and Spearman´s rho (r_s) with corresponding *p*-value (* for p < .05) and 95% confidence interval via bootstrapping (CI) for differences between T_0 and T_1 for each measurement tool. Additionally, non-wear time of the accelerometer in minutes per week (min/wk) is displayed.

	n	T_0 Mean (SD)	T_1 Mean (SD)	r_s (*p*-value) [CI]
**MPA (min/wk)**				
Acc 10	28	646.42 (271.37)	609.73 (317.54)	.792 (<.001*) [.507-.926]
Acc 60	28	439.96 (221.47)	428.33 (274.48)	.797 (<.001*) [.532-.917]
Diary	27	282.33 (524.86)	215.52 (336.09)	.278 (.161) [-.134-.624]
IPAQ	27	810.93 (832.75)	695.00 (799.19)	.502 (.008*) [.105-.766]
**VPA (min/wk)**				
Acc 10	28	48.97 (61.18)	52.00 (62.94)	.572 (.001*) [.186-.871]
Acc 60	28	33.59 (61.70)	28.64 (50.31)	.668 (<.001*) [.288-.871]
Diary	27	28.11 (59.35)	19.52 (42.22)	.292 (.140) [-.149-.684]
IPAQ	28	122.32 (277.63)	154.29 (281.89)	.153 (.437) [-.295-.548]
Non-wear time (min/wk)	28	3965.54 (422.55)	4179.80 (582.05)	-

Table S2. Descriptive data of moderate (MPA) and vigorous (VPA) physical activity of children. Displayed are the number of participants (n), means and standard deviations (SD) at two measurements at least three weeks apart (T_0 and T_1) for the parameters accelerometry with 60 seconds epoch length (Acc 60) and 10 seconds epoch length (Acc 10), physical activity diary (diary), and Spearman´s rho (r_s) with corresponding *p*-value (* for p < .05) and 95% confidence interval via bootstrapping (CI) for differences between T_0 and T_1 for each measurement tool. Additionally, non-wear time of the accelerometer in minutes per week (min/wk) is displayed.

	n	T_0 Mean (SD)	T_1 Mean (SD)	r_s (*p*-value) [CI]
**MPA (min/wk)**				
Acc 10	24	652.98 (246.81)	588.00 (239.68)	.608 (.002*) [.160-.865]
Acc 60	24	574.21 (267.41)	496.90 (250.95)	.664 (<.001*) [.300-.899]
Diary	30	260.23 (258.54)	182.00 (180.63)	.379 (.039*) [.001-.669]
**VPA (min/wk)**				
Acc 10	24	87.89 (79.19)	71.61 (48.45)	.759 (<.001*) [.536-.890]
Acc 60	24	58.46 (73.38)	46.04 (39.40)	.604 (.002*) [.261-.824]

Diary	30	102.53 (144.59)	81.00 (148.28)	.485 (.007*) [.163-.752]
Non-wear time (min/wk)	24	4559.10 (848.50)	4929.41 (695.22)	-

Table S3. Validity between all measurement methods for moderate (MPA) and vigorous (VPA) physical activity of adults and children at T_0. Displayed are the parameters accelerometry with 10 seconds epoch length (Acc 10) and 60 seconds epoch length (Acc 60), physical activity diary (diary), the International Physical Activity Questionnaire (IPAQ), and Spearman´s rho (*r*) with corresponding *p*-value (* for p < .05) and confidence interval via bootstrapping (CI) for differences between each measurement tool.

	**T_0 *r* (*p*-value) [CI]**				
	**Adults**			**Children**	
**MPA (min/wk)**					
	Acc 10	Acc 60	Diary	Acc 10	Acc 60
Acc 60	.974 (<.001*) [.901 - .990]	-	-	.981 (<.001*) [.928-.994]	-
Diary	.209 (.259) [-.107 - .557]	.213 (.250) [-.189 - .551]	-	.232 (.234) [-.192-.603]	.258 (.184) [-.148-.601]
IPAQ	.277 (.132) [-.134 - .629]	.247 (.180) [-.158 - .599]	.265 (.157) [-.102 - .564]	-	-
**VPA (min/wk)**					
Acc 60	.904 (<.001*) [.756 - .969]	-	-	.972 (<.001*) [.905-.992]	-
Diary	.146 (.432) [-.050 - .529]	.062 (.739) [-.354 - .520]	-	.173 (.379) [-.224-.550]	.222 (.255) [-.215-.567]
IPAQ	.379 (.039*) [-.010 - .691]	.313 (.092) [-.081 - .639]	.413 (.026*) [.025 - .715]	-	-

Table S4. Validity between all measurement methods for moderate (MPA) and vigorous (VPA) physical activity of adults and children at T_1. Displayed are the parameters accelerometry with 10 seconds epoch length (Acc 10) and 60 seconds epoch length (Acc 60), physical activity diary (diary), the International Physical Activity Questionnaire (IPAQ), and Spearman´s rho (*r*) with corresponding *p*-value for differences between each measurement tool.

	**T_1 *r* (*p*-value) [95% CI]**				
	**Adults**			**Children**	
**MPA (min/wk)**					
	Acc 10	Acc 60	Diary	Acc 10	Acc 60
Acc 60	.955 (<.001*) [.868 - .990]	-	-	.967 (<.001*) [.887-.990]	-
Diary	-.237 (.244) [-.602 - .177]	-.304 (.131) [-.648 - .131]	-	-.100 (.625) [-.501-.361]	-.056 (.786) [-.437-.377]
IPAQ	.306 (.129) [-.120 - .658]	.236 (.246) [-.222 - .580]	.283 (.170) [-.171 - .660]	-	-
**VPA (min/wk)**					
Acc 60	.925 (<.001*) [.827 - .966]	-	-	.916 (<.001*) [.732-.982]	-
Diary	.255 (.209) [-.147 - .578]	.322 (.108) [-.106 - .639]	-	.204 (.317) [-.225-.543]	.181 (.376) [-.222-.566]
IPAQ	.275 (.165)	.178 (.374)	.349 (.074)	-	-

	[-.145 - .666]	[-.272 - .529]	[.030 - .659]
